# Endocarditis in Liver Transplant Recipients: A Systematic Review

**DOI:** 10.3390/jcm10122660

**Published:** 2021-06-16

**Authors:** Petros Ioannou, Konstantinos Alexakis, Diamantis P Kofteridis

**Affiliations:** Department of Internal Medicine & Infectious Diseases, University Hospital of Heraklion, 71110 Heraklion, Crete, Greece; a.konstantin91@gmail.com (K.A.); kofteridisd@hotmail.com (D.P.K.)

**Keywords:** liver transplant, endocarditis, systematic review

## Abstract

Infective Endocarditis (IE) is associated with significant mortality. Interestingly, IE in patients with liver transplantation has not been adequately described. The aim of this review was to systematically review all published cases of IE in liver transplant recipients and describe their epidemiology, microbiology, clinical characteristics, treatment and outcomes. A systematic review of PubMed, Scopus and Cochrane Library (through 2 January 2021) for studies providing epidemiological, clinical, microbiological, treatment data and outcomes of IE in liver transplant recipients was conducted. A total of 39 studies, containing data for 62 patients, were included in the analysis. The most common causative pathogens were gram-positive microorganisms in 69.4%, fungi in 25.8%, and gram-negative microorganisms in 9.7% of cases, while in 9.3% IE was culture-negative. The aortic valve was the most commonly infected valve followed by mitral, tricuspid and the pulmonary valve. Aminoglycosides, vancomycin and aminopenicillins were the most commonly used antimicrobials, and surgical management was performed in half of the cases. Clinical cure was noted in 57.4%, while overall mortality was 43.5%. To conclude, this systematic review thoroughly describes IE in liver transplant recipients and provides information on epidemiology, clinical presentation, treatment and outcomes.

## 1. Introduction

Infective Endocarditis (IE) is a rare disease that carries significant morbidity and mortality [1,2]. Bacteremia, a predisposing factor for developing IE, may occur more frequently in immunosuppressed individuals, such as patients who have undergone solid organ transplantation [3]. Interestingly, IE in liver transplant recipients has not been adequately described, even though there are isolated reports of such cases and there are large registries of patients with infective endocarditis which have attempted to characterize these patients, and yielded only a few patients at a time [4,5,6].

The aim of this study was to systematically review all cases of IE in liver transplant recipients in the literature and describe their epidemiology, microbiology, clinical characteristics, treatment and outcomes.

## 2. Materials and Methods

### 2.1. Data Search

For this review, we adopted the Meta-analysis of Observational Studies in Epidemiology (MOOSE) guidelines [7]. Eligible studies were identified through search of PubMed, Scopus and Cochrane Library with the following text-words: (liver OR hepatic) AND (transplant OR transplantation OR graft) AND endocarditis. Day of first search was 26 November 2020 and day of last search was 2 January 2021.

### 2.2. Study Selection

Studies were included in the analysis if they met the following criteria: (1) published in English; (2) reporting data on patients’ clinical characteristics, microbiology, treatment and outcomes. Studies with the following criteria were excluded: (1) secondary research papers (e.g., reviews), editorials and papers not reporting results on primary research; (2) studies not in humans; (3) studies in non-transplanted patients; (4) studies not in English. Two investigators (P.I., K.A.) using Abstrackr [8] independently reviewed the titles and abstracts of the resulting references and then retrieved and rescreened the full text publications of potentially relevant articles. Study selection was based on consensus. Reference lists of included studies were searched for relevant articles. In cases where the investigators were unable to access a full text publication, attempts were made to communicate with the study authors in order to kindly provide the full text.

### 2.3. Study Outcomes

The primary study outcomes of this study were to record: (a) epidemiological characteristics of patients with liver transplantation and IE and (b) data on overall and IE-specific mortality, while secondary outcomes were to record: (a) the microbiological data of IE, (b) the clinical characteristics of the patients and, (c) their treatment. Another endpoint was the identification of independent risk factors for mortality by these infections.

### 2.4. Data Extraction and Definitions

Data from each eligible study were extracted by two investigators (P.I., K.A.). The extracted data included study type, year of publication and country; patient demographic data (age and gender); patient’s relevant medical history (cause of liver transplant, time after transplantation, presence of a prosthetic cardiac valve); infection data and microbiology (infection site, isolated bacterial strains, presence of complications, presence of embolic phenomena); treatment administered for IE; and outcomes (i.e.., cure or death). Relation of death to the index infection was reported according to the study authors. Diagnosis of IE was confirmed by the investigators based on the information provided by the authors and the modified Dukes’ criteria if the diagnosis was possible (at least 1 major and 1 minor criterion or at least 3 minor criteria) or if pathological data established a diagnosis of IE [9]. The complications recorded included any organ dysfunction or clinical deterioration that was considered by the authors to be related to the IE. The quality of evidence of the outcomes of included studies was assessed using the Grading of Recommendations Assessment, Development and Evaluation (GRADE) [10].

### 2.5. Statistical Analysis

Data are presented as number (%) for categorical variables and median (interquartile range, IQR) or mean (±standard deviation, SD) for continuous variables. A univariate logistic regression analysis was conducted to identify factors associated with all-cause mortality of patients with liver transplantation and IE. Furthermore, we performed a univariate logistic regression analysis in order to identify an association between having received a biologic agent for induction of immunosuppression and developing IE by fungi. The above-mentioned statistics were calculated with GraphPad Prism 6.0 (GraphPad Software, Inc., San Diego, CA, USA).

A multivariate logistic regression analysis was conducted to evaluate the effect of factors that were previously identified in the univariate analysis model with a p-value lower than 0.05. Multivariate analysis was performed using SPSS version 23.0 (IBM Corp., Armonk, NY, USA).

## 3. Results

### 3.1. Literature Search

**Figure 1:**
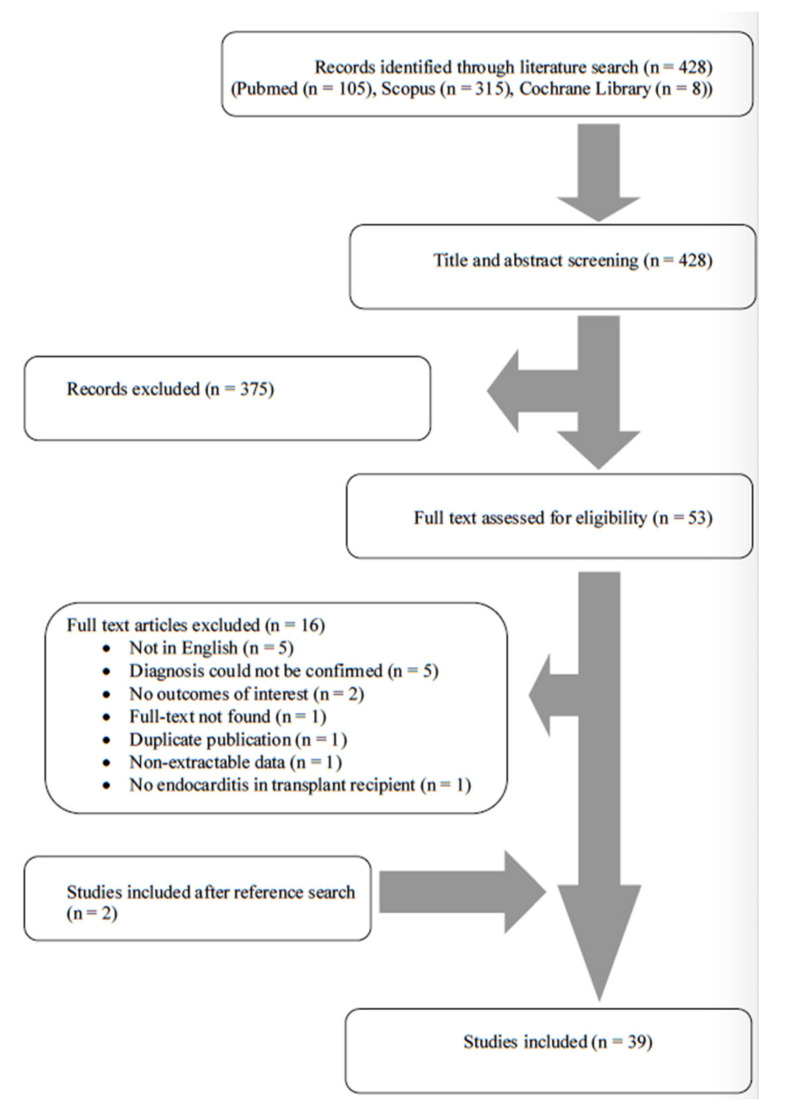
Flow diagram of study inclusion.

A total of 428 articles from PubMed, Scopus and Cochrane Library were screened. After reviewing the titles and abstracts, 53 articles were selected for full-text review. From these studies, 16 were excluded from the review: five studies were not in English, in five studies the diagnosis of IE could not be confirmed with the Dukes criteria, two studies had no outcomes of interest, one full text could not be found, one study was a duplicate, in one study the data were non-extractable and in one study there was no IE in the liver transplant recipient. Additionally, two studies were included after reference search of the abovementioned studies. Finally, 39 studies met the present study’s inclusion criteria [6,11,12,13,14,15,16,17,18,19,20,21,22,23,24,25,26,27,28,29,30,31,32,33,34,35,36,37,38,39,40,41,42,43,44,45,46,47,48]. Additional information was kindly provided by the corresponding authors of two studies [40,48]. The review process is graphically presented in Figure 1.

### 3.2. Included Studies’ Characteristics

Supplementary Table S1 summarizes the characteristics of included studies. The 39 studies that were included in the present analysis involved 62 patients in total. Among them, 20 were conducted in North and South America, 12 in Europe, six in Asia, and one in Oceania. There were 28 case reports, nine retrospective studies and two prospective studies, thus, the overall quality of the evidence that contributed to this systematic review was rated as low to very low [10].

### 3.3. Characteristics of IE in Liver Transplant Recipients

Table 1 shows the characteristics of the patients with IE and liver transplantation. Age of patients ranged from 0.67 to 78 years, the median age was 53.5 years, and 71% (n = 44/62 patients) were male. Time from transplantation ranged from days to 17 years, and median was 3 months. Immunosuppression included glucocorticoids in 56.7% (n = 17/30 patients with available data), tacrolimus only in 31% (n = 9/29), combination of mycophenolate mofetil with tacrolimus in 24.1% (n = 7), cyclosporine only in 13.8% (n = 4), combination of mycophenolate mofetil with cyclosporine in 13.8% (n = 4), combination of cyclosporine and azathioprine in 10.3% (n = 3), combination of rapamycin with mycophenolate mofetil in 3.4% (n = 1) and everolimus in 3.4% (n = 1). Importantly, 21.4% (6/28) of patients had received a biologic agent for induction of immunosuppression. When data were available, the commonest cause of cirrhosis that led to the liver transplantation was hepatitis C virus (HCV) infection in 21.9% (n = 7/32), hepatitis B virus (HBV) infection in 12.5% (n = 4), primary biliary cirrhosis in 12.5% (n = 4), ethanol use in 12.5% (n = 4), cryptogenic in 9.4% (n = 3), non-alcoholic steatohepatitis in 9.4% (n = 3), combination of HCV infection and ethanol use in 6.3% (n = 2), primary sclerosing cholangitis in 6.3% (n = 2), and hepatic cholangiocarcinoma, hemochromatosis and biliary atresia in 3.1% (n = 1) each. A prosthetic valve was present in 4.2% (n = 2/48), while no patient had an intracardiac device (intracardiac defibrillator or pacemaker) prior to development of IE.

Clinical presentation of patients with liver transplantation and IE is shown in Table 1. The most common sites of infection were the lower respiratory tract in 9.7% (n = 6/62 patients), the central nervous system in 6.5% (n = 4), the liver in 4.8% (n = 3), the bones in 4.8% (n = 3), septic arthritis in 4.8% (n = 3) and the peritoneal cavity in 3.2% (n = 2). The most common site of infection was the aortic valve in 46.7% (n = 28/60), the mitral valve in 41.2% (n = 25), the tricuspid valve in 18.6% (n = 11), the pulmonary valve in 6.7% (n = 4), while in 13.3% (n = 8) no valve was infected, but the infection was at the mural endocardium. Multiple valves were infected in 21.7% (n = 13). Diagnosis was set with a transthoracic heart ultrasound in 40% (n = 14/35), a transesophageal heart ultrasound in 28.6% (n = 10), a valve culture in 18.9% (n = 7/37), and at autopsy in 28.3% (n = 17/60). Diagnosis was eventually confirmed by this study’s authors through pathological data in 25.8% (16/62) of patients, while in the other patients (74.2%, 46/62), the diagnosis was confirmed through the Dukes criteria, with, 71% (44/62) of patients fulfilling two major criteria, 1.6% (one patient) fulfilling one major and two minor criteria and 1.6% (one patient) fulfilling one major and one minor criterion.

### 3.4. Microbiology of IE in Liver Transplant Recipients

**Figure 2:**
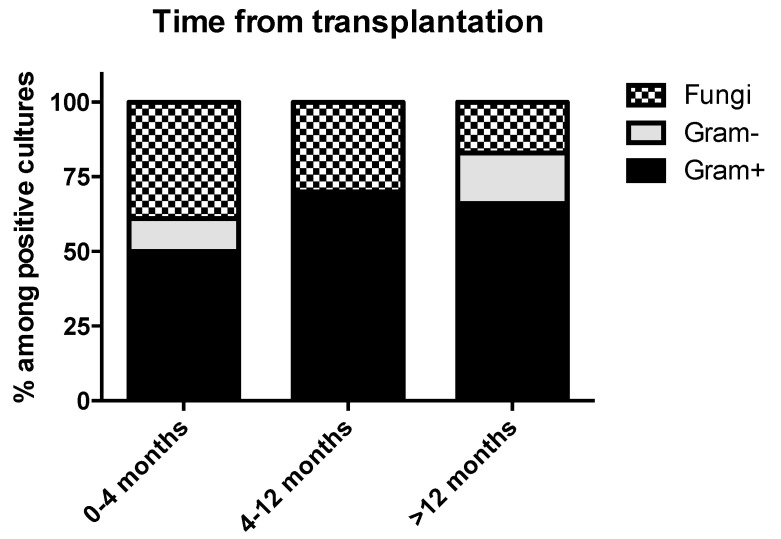
Distribution of pathogens causing Infective Endocarditis with respect to time from liver transplantation. Bars show percentages among 47 patients with available data. Gram-: Gram-negative microorganisms; Gram+: Gram-positive microorganisms.

The most commonly identified microorganisms were gram-positives in 57.4% (n = 66/115), namely *Enterococci* in 26.1% (n = 30), *Streptococci* in 14.8% (n = 17), and *Staphylococci* in 13% (n = 15), gram-negatives in 14.8% (n = 17), fungi in 20% (n = 23), while in 18.9% (n = 21/110) IE was culture negative. IE was polymicrobial in 2.6% (n = 3). Supplementary Table S2 summarizes the microbiology of IE in patients with liver transplantation, while Figure 2 shows the distribution of pathogens causing IE, depending on the time after transplantation. Importantly, a univariate logistic regression analysis of having received a biologic agent for induction of immunosuppression and developing fungal IE revealed a statistically significant positive association (slope = 0.4394 ± 0.2059, *r^2^* = 0.149, *p* = 0.0425).

### 3.5. Treatment and Outcomes of IE in Liver Transplant Recipients

For the treatment of IE, antimicrobial therapy included combination of antimicrobials in the vast majority of cases, with antimicrobials used being aminoglycosides in 32.6% (n = 15/46), vancomycin in 30.4% (n = 14), aminopenicillins in 23.9% (n = 11), cephalosporins and rifampicin in 13% (n = 6) each, daptomycin and anti-staphylococcal penicillins in 8.7% (n = 4) each, carbapenems in 6.5% (n = 3), linezolid, quinolones and tigecycline in 4.3% (n = 2) each, and antipseudomonal penicillins, and co-trimoxazole in 2.2% (n = 1) each. For fungal IE, amphotericin B was used in 50% (n = 8/16), voriconazole and caspofungin in 18.8% (n = 3) each, and fluconazole, itraconazole, and 5-fluocytosine in 6.3% (n = 1) each. Surgical management was performed in 50.9% (n = 27/53). Duration of treatment ranged from 2 to 136 weeks, with a median duration of 6 weeks. Treatment and outcomes of IE in patients with liver transplantation can be seen in detail in Supplementary Table S1.

### 3.6. Statistical Analysis of IE in Patients with Liver Transplantation

We performed a univariate logistic regression analysis in order to identify any association between gender, age, having a prosthetic cardiac valve, being an IVDU, having a CVC, having ESRD on hemodialysis, years after transplantation, being on corticosteroids, having a transplant rejection or graft failure or liver failure during hospitalization, having a polymicrobial IE, having IE by gram-positive or gram-negative microorganisms or fungi, having IE by *S. aureus* or *Enterococcus* spp. or coagulase negative staphylococci, having culture-negative IE, having IE at the mitral, the aortic or the tricuspid valve, or in multiple valves, presentation with sepsis, shock, heart failure, embolic or immunologic phenomena and having a surgery along with antimicrobial treatment, with overall mortality. The analysis identified a statistically significant positive association of overall mortality with liver failure (*p* = 0.0021), graft failure (*p* = 0.0174) having IE by a fungus (*p* = 0.0003), having culture-negative IE (*p* = 0.004) and presenting with shock (*p* = 0.0031) and a statistically significant negative association of overall mortality with having an IE by gram-positive microorganisms in general (*p* = 0.039) and *E. faecalis* specifically (0.0445) as well as having surgery along with antimicrobial treatment (*p* = 0.0001). Thus, a multivariate logistic regression analysis was performed with the abovementioned parameters, but did not recognize any independent factor associated with overall mortality.

## 4. Discussion

In recent decades, the development of liver transplantation both in terms of surgical techniques, as well as in terms of post-operative pharmacological care, has led to prolongation of survival and improvement of quality of life for patients with end-stage chronic liver disease [49,50]. Infections in patients with liver transplantation pose an important cause of morbidity and mortality [51,52,53,54]. However, even though several different infections have been described in these patients, there are scarce reports of IE in patients with liver transplantation [4]. To the best of our knowledge, this is the first study to systematically review IE in patients with liver transplantation, and provide detailed information on epidemiology, clinical characteristics, microbiology, treatment and outcomes.

The median age of patients with liver transplantation and IE was lower than the age in other cohorts with patients with IE, where mean age is around 70 years [55,56,57]. However, the age is similar to that of patients with end-stage liver disease, as well as in patients with IE and kidney transplantation [58,59]. This is also in line with the literature, as in a recent study patients with solid organ transplantation and IE had a lower age compared with patients with IE but without solid organ transplantation [4]. Male predominance was noted in our population, as in other studies with IE in the general population and in patients with IE and transplantation [56,57,59]. Importantly, the percentage of patients with liver transplantation and IE who had a prosthetic valve was quite lower (less than 5%) than that of patients with IE from the general population, which can be up to 50% [55,56,57]. However, in another systematic review of patients with IE and kidney transplant recipients, a similar percentage of patients with a prosthetic valve was found [59]. In the population of our study, blood culture-negative IE had a rate similar to that in the general population and to that of patients with IE and kidney transplantation [55,56,57,59].

In studies of IE in the general population, the most frequently identified pathogens were *Staphylococcus*, *Streptococcus* and *Enterococcus* microorganisms [55,56,57]. In the present systematic review, the microbiology of IE in liver transplant recipients was different, with *Enterococcus* and fungi being the most frequent pathogens. Interestingly, in a recent multi-center study describing nosocomial IE, *Enterococcus* emerges as the most frequent pathogen of IE [57]. On the other hand, in another recent systematic review of patients with kidney transplantation, *Enterococcus* was again identified as the most commonly identified pathogen causing IE [59]. This seems to foster the assumption that patients with IE and solid organ transplantation may have a microbiology that resembles that of the nosocomial IE, since this population has a very close relationship with the healthcare system. This, however, is in contrast with the findings of a recent study showing that patients with solid organ transplantation may be more likely to have IE of staphylococcal etiology [4]. Furthermore, the microbiology of patients with IE and liver transplantation also differs from the microbiology of patients with IE and liver cirrhosis, where the most commonly identified pathogens are *Staphylococcus aureus* and *Streptococcus* spp., with *Enterococcus* being the third most commonly identified pathogen [58], implying that the status of transplantation may be associated with increased occurrence of IE by *Enterococcus* spp. On the other hand, patients with liver transplantation may have recurrent hospitalizations, prolonged stays in the hospital or the intensive care unit, multiple indwelling vascular catheters, need for hemodialysis, and multiple courses of antimicrobial treatment for infections such as bacteremia, cholangitis, spontaneous bacterial peritonitis, that impact their risk of infection and colonization with nosocomial and multi-drug resistant organisms. Furthermore, after transplantation, complications such as anastomotic leak and fluid collection may also impact microbiology. *Enterococcus* spp. are important pathogens of the gastrointestinal tract that could be implicated in these processes, and this could partially explain the observed difference in microbiology. Finally, the type of antimicrobial prophylaxis administered post-operatively could also have impacted the noted differences in the microbiology of IE, even though this information was not readily available in the included studies, and thus, is not presented in this systematic review.

In the general population, *Candida* spp. is the most common fungal etiology of IE, even though fungal IE is quite rare [60,61]. However, in this study, the most common fungal cause of IE in patients with liver transplantation was *Aspergillus* spp. and the same is the case in other patients with organ transplantation [59,62]. Since the number of fungal IE in this study was small, these results should be addressed with caution. Even so, the occurrence of invasive aspergillosis in liver transplant recipients (as well as in other patients with transplantation) may not be an unexpected finding, as these patients have acquired defects in their cellular immunity due to the fact that they require immunosuppressive treatment in order to avoid rejection of the transplants. On the other hand, some centers are using antifungal prophylaxis with fluconazole or echinocandins, and this may have affected the microbiology of fungal infections, thus partially explaining the predominance of *Aspergillus* fungal IE in this population, since antifungal prophylaxis is highly active against *Candida* spp. [63,64]. Importantly, as in patients with kidney transplantation, fungal IE was more frequent during the first months after transplantation, while the frequency was decreased as time from transplantation increased. This may be directly associated with the intensity of induction immunosuppression. For example, in this study we identified a statistically significant positive association between use of a biologic agent for induction of immunosuppression and development of fungal IE.

Patients with liver transplantation were more likely to present with fever and sepsis compared to patients with kidney transplantation, but were as likely to present with embolic phenomena and heart failure [59]. These ratios were higher than those noted in the general population with IE [55,56]. Finally, mortality in patients with liver transplantation was higher than in the general population and was comparable to that of patients with kidney transplantation [55,56,59]. This could partially reflect the very high mortality of fungal IE, which contributes significantly to this patient population.

The present systematic review has some limitations that should be mentioned. First of all, it mainly consists of case reports and case series. Thus, results should be read cautiously, as case reports may describe unusual presentations, implying that usual presentations may be underrepresented in this systematic review. This could have affected the above-mentioned microbiology; thus, fungi could have been overrepresented in this systematic review. However, this is the only methodology that could be used to systematically study IE in liver transplant recipients. If case reports, case series and studies describing less than four patients were excluded, there would be only three studies left for inclusion, with only 25 patients being analyzed [17,25,33]. This could also lead to misrepresentation of microbiological data. Thus, we opted to include all well-described and informative cases of patients with IE in patients with liver transplantation. Finally, excluding non-English articles may have impacted the inclusion of articles from Asia not written in English, where liver transplantation for HBV and associated hepatocellular carcinoma are more common, and this may limit the applicability of our findings.

## 5. Conclusions

In conclusion, this study describes the epidemiology, clinical characteristics, microbiology, treatment and outcomes of IE in patients with liver transplantation. IE in these patients carries significant mortality, while *Enterococcus* and fungi seem to be important pathogens. Physicians looking after patients with liver transplantation should be familiarized with these infections, as they are associated with significant morbidity and mortality.

## Figures and Tables

**Table 1:** Characteristics of 62 patients with liver transplantation and Infective Endocarditis. Values show cases among patients with available data.

Characteristic	Value
Male, n (%)	44/61 (71%)
Age, median (IQR) in years	53.5 (41–60)
Years of transplantation before IE, median (IQR)	0.25 (0.05–17)
CVC, n (%)	8/46 (17.4%)
Prosthetic valve, n (%)	2/48 (4.2%)
IVDU, n (%)	1/57 (1.8%)
Clinical characteristics	
Feverish, n (%)	23/23 (100%)
Sepsis, n (%)	13/18 (72.2%)
Embolic phenomena, n (%)	15/32 (46.9%)
Heart failure, n (%)	6/21 (28.6%)
Liver failure, n (%)	8/33 (24.2%)
Graft failure, n (%)	6/32 (18.8%)
Shock, n (%)	4/24 (16.7%)
Transplant rejection, n (%)	3/31 (9.7%)
Immunologic phenomena, n (%)	1/15 (6.7%)
Paravalvular abscess, n (%)	1/17 (5.9%)
Outcomes	
Clinical cure, n (%)	35/61 (57.4%)
Deaths due to the infection, n (%)	25/62 (40.3%)
Deaths overall, n (%)	27/62 (43.5%)
Time of follow-up in months, median (IQR)	12 (7–22)

CVC: central venous catheter; IE: Infective Endocarditis; IVDU: intravenous drug use; IQR: intraquartile range.

## Data Availability

The data presented in this study are available on request from the corresponding author.

## Supplementary Materials

The following are available online at https://www.mdpi.com/article/10.3390/jcm10122660/s1, Table S1: Included studies’ characteristics, Table S2: Microbiology of Infective Endocarditis in liver transplant recipients. Values show cases among patients with available data.
