# Supplementary material for: Endocarditis in Liver Transplant Recipients: A Systematic Review
Source: J Clin Med. 2021 Jun 16;10(12):2660. doi: 10.3390/jcm10122660 (PMC8235265; doi:10.3390/jcm10122660)
Supplement: Supplementary file 1 [file jcm-10-02660-s001.zip › jcm-1252185-supplementary/@Supplementary Table 1.pdf]

Supplementary Table 1. Included studies' characteristics

Study, year published	Number of patients	Age (years)	Gender	Site of infection, n (%)	Microbiology of infection, n (%)	Treatment administered	Infection Outcomes, n (%)
Woods et al, 1989 [11]	1	19	Male	TrV 1 (100)	*Aspergillus flavus* 1 (100)	Amphotericin B 1 (100)	Clinical cure[a] 0 (0) Overall mortality 1 (100) IE-specific mortality 1 (100)
Estol et al, 1991 [12]	1	55	Female	AoV 1 (100)	*Staphylococcus aureus* 1 (100)	NR 1 (100)	Clinical cure 0 (0)

							Overall mortality 1 (100) IE-specific mortality 1 (100)
Welty et al, 1992 [13]	1	53	Male	PV 1 (100)	*Pseudallescheria boydii* 1 (100)	No antifungals 1 (100)	Clinical cure 0 (0) Overall mortality 1 (100) IE-specific mortality 1 (100)

David et al, 1992 [14]	1	56	Female	AoV 1 (100)	*Corynebacterium jeikeium* 1 (100)	Cephalosporin 1 (100)<br>Vancomycin 1 (100)<br>Surgical management 1 (100)	Clinical cure 1 (100)<br>Overall mortality 0 (0)
Miga et al, 1996 [15]	1	1.33	Male	TrV 1 (100)	*Staphylococcus aureus* 1 (100)	NR 1 (100)<br>Surgical management 1 (100)	Clinical cure 1 (100)<br>Overall mortality 0 (0)
Heath et al, 1997 [16]	1	45	Male	MV 1 (100)<br>TrV 1 (100)	*Phaeoacremonium parasiticum* 1 (100)	NR 1 (100)	Clinical cure 0 (0)<br>Overall

							mortality 1 (100)<br>IE-specific mortality 1 (100)
Paterson et al, 1997 [17]	10	Median 55<br>Range (39-61)	9 male<br>1 female	AoV 5 (50)<br>Mural 3 (30)<br>MV 2 (20)<br>TrV 1 (10)<br>PV 1 (10)	*Aspergillus fumigatus* 4 (40)<br>*Staphylococcus aureus* 4 (40)<br>*Enterococcus faecium* 1 (10)<br>*Enterococcus faecalis* 1 (10)	Vancomycin 5 (50)<br>Aminoglycoside 3 (30)<br>Amphotericin B 3 (30)<br>Rifampicin 2 (20)<br>Quinolone 1 (10)<br>No antimicrobial used 1 (10)	Clinical cure 4 (40)<br>Overall mortality 6 (60)<br>IE-specific mortality 6 (60)

						Anti-staphylococcal penicillin 1 (10)<br>Surgical management 3 (30)	
Hearn et al, 1999 [18]	1	59	Female	PV 1 (100)	*Staphylococcus aureus* 1 (100)	Vancomycin 1 (100)<br>Rifampicin 1 (100)<br>Surgical management 1 (100)	Clinical cure 1 (100)<br>Overall mortality 0 (0)
Avery et al, 1999 [19]	1	41	Female	TrV 1 (100)	*Listeria monocytogenes* 1	Aminopenicillin 1 (100)	Clinical cure 1 (100)

					(100)	Aminoglycoside 1 (100)	Overall mortality 0 (0)
Lorf et al, 1999 [20]	1	60	Male	Mural 1 (100)	*Aspergillus* spp. 1 (100)	NR 1 (100)	Clinical cure 0 (0)<br>Overall mortality 1 (100)<br>IE-specific mortality 1 (100)
Moreira et al, 2000 [21]	1	64	Male	AoV 1 (100)<br>MV 1 (100)	*Cutibacterium (Propionibacterium) acnes* 1 (100)	Vancomycin 1 (100)<br>Aminoglycoside 1 (100)	Clinical cure 1 (100)<br>Overall mortality 0 (0)

						Surgical management 1 (100)	
Romagnuolo et al, 2000 [22]	1	37	Male	Mural 1 (100)	*Aspergillus* spp. 1 (100)	Amphotericin B 1 (100)	Clinical cure 1 (0)<br>Overall mortality 1 (100)<br>IE-specific mortality 1 (100)
Gotsman et al, 2003 [23]	1	33	Male	TrV 1 (100)	*Staphylococcus aureus* 1 (100)	Vancomycin 1 (100)<br>Aminoglycoside 1	Clinical cure 1 (100)<br>Overall

						(100)<br>Surgical management 1 (100)	mortality 0 (0)
Haddad et al, 2004 [24]	1	48	Female	MV 1 (100)	*Staphylococcus aureus* 1 (100)	Anti-staphylococcal penicillin 1 (100)<br>Rifampicin 1 (100)	Clinical cure 0 (0)<br>Overall mortality 1 (100)<br>IE-specific mortality 1 (100)
Ruttman et al, 2005 [25]	7	Median 53<br>Range (40-60)	6 male<br>1 female	AoV 7 (100)<br>MV 4 (57.1)	Coagulase-negative *Staphylococcus* 3	NR 7 (100)<br>Surgical	Clinical cure 2 (28.6)

					(42.9)<br>*Staphylococcus aureus* 2 (28.6)<br>*Aspergillus* spp. 2 (28.6)<br>*Enterococcus* spp. 2 (28.6)<br>*Enterobacter* spp. 1 (14.3)	management 2 (28.6)	Overall mortality 5 (71.4)<br>IE-specific mortality 5 (71.4)
Salamon et al, 2006 [26]	1	26	Female	TrV 1 (100)<br>PV 1 (100)	*Candida albicans* 1 (100)	Fluconazole 1 (100)<br>5-fluocytosine 1 (100)<br>Caspofungin 1 (100)	Clinical cure 1 (100)<br>Overall mortality 0 (0)

						Surgical management 1 (100)	
Goegele et al, 2007 [27]	1	45	Male	AoV 1 (100)	Coagulase-negative *Staphylococcus* 1 (100)<br>*Klebsiella terrigena* 1 (100)	Carbapenem 1 (100)<br>Vancomycin 1 (100)<br>Linezolid 1 (100)	Clinical cure 0 (0)<br>Overall mortality 1 (100)<br>IE-specific mortality 1 (100)
Vianna et al, 2007 [28]	1	41	Male	Mural 1 (100)	*Aspergillus fumigatus* 1 (100)	Amphotericin B 1 (100)<br>Voriconazole 1	Clinical cure 1 (100)<br>Overall

						(100)<br>Caspofungin 1 (100)<br>Surgical management 1 (100)	mortality 0 (0)
Kumar et al, 2007 [29]	1	58	Male	AoV 1 (100)	*Staphylococcus schleiferi* 1 (100)	Vancomycin 1 (100)<br>Aminoglycoside 1 (100)<br>Rifampicin 1 (100)	Clinical cure NR (100)<br>Overall mortality 1 (100)<br>IE-specific mortality 0 (0)
Goegele et al,	1	53	Male	AoV 1 (100)	Coagulase-negative	Teicoplanin 1	Clinical cure 0

2007 [30]					*Staphylococcus* 1 (100)<br>*Klebsiella terrigena* 1 (100)	(100)	(0)<br>Overall mortality 1 (100)<br>IE-specific mortality 1 (100)
Mourier et al, 2009 [31]	1	0.67	Male	MV 1 (100)	*Aspergillus* spp. 1 (100)	Amphotericin B 1 (100)<br>Voriconazole 1 (100)	Clinical cure 1 (100)<br>Overall mortality 0 (0)
Vernadaki et al, 2009 [32]	1	55	Male	AoV 1 (100)<br>TrV 1 (100)	*Staphylococcus aureus* 1 (100)	Daptomycin 1 (100)<br>Surgical	Clinical cure 1 (100)<br>Overall

						management 1 (100)	mortality 0 (0)
Forrest et al, 2011 [33]	8	Median 46<br>Range (26-74)	3 male<br>5 female	MV 6 (75)<br>AoV 2 (25)<br>TrV 1 (12.5)	*Enterococcus faecalis* 6 (75)<br>*Enterococcus faecium* 2 (25)	Aminoglycoside 7 (87.5)<br>Anti-staphylococcal penicillin 6 (75)<br>Daptomycin 2 (25)<br>Tigecycline 1 (12.5)<br>Surgical Management 5 (62.5)	Clinical cure 6 (75)<br>Overall mortality 2 (25)<br>IE-specific mortality 2 (25)
Walter et al,	1	63	Male	MV 1 (100)	*Staphylococcus*	NR 1 (100)	Clinical cure 0

2011 [34]					*aureus* 1 (100)	Surgical management 1 (100)	(0) Overall mortality 1 (100) IE-specific mortality 0 (0)
Castelli et al, 2011 [35]	1	36	Male	MV 1 (100)	*Corynebacterium* spp. 1 (100) *Nocardia* spp. 1 (100)	Vancomycin 1 (100) Co-trimoxazole 1 (100) Surgical management 1 (100)	Clinical cure 1 (100) Overall mortality 0 (0)
Mrzljak et al,	1	63	Male	MV 1 (100)	*Staphylococcus*	Anti-	Clinical cure 1

2012 [36]					*lugdunensis* 1 (100)	staphylococcal penicillin 1 (100)<br>Vancomycin 1 (100)<br>Surgical management 1 (100)	(100)<br>Overall mortality 0 (0)
George et al, 2013 [37]	1	51	Male	MV 1 (100)	*Escherichia coli* 1 (100)	Carbapenem 1 (100)<br>Tigecycline 1 (100)	Clinical cure 1 (100)<br>Overall mortality 0 (0)
Belvisi et al, [38]	1	61	Female	MV 1 (100)	*Streptococcus pneumoniae* 1 (100)	Linezolid 1 (100)<br>Cephalosporin 1 (100)	Clinical cure 1 (100)<br>Overall

							mortality 0 (0)
Borde et al, 2013 [39]	1	36	Female	AoV 1 (100)	*Streptococcus viridans* 1 (100)	Cephalosporin 1 (100)<br>Surgical management 1 (100)	Clinical cure 1 (100)<br>Overall mortality 0 (0)
Fernández-Hidalgo [40]	2	56, 78	2 male	MV 1 (50)<br>AoV 1 (50)	*Enterococcus faecalis* 2 (100)	Cephalosporin 2 (100)<br>Aminopenicillin 2 (100)<br>Surgical management 1 (50)	Clinical cure 2 (100)<br>Overall mortality 0 (0)
Dahya et al	1	62	Male	AoV 1 (100)	*Peptostreptococcus*	Vancomycin 1	Clinical cure 1

[41]					spp. 1 (100)	(100)<br>Cephalosporin 1 (100)<br>Surgical management 1 (100)	(100)<br>Overall mortality 0 (0)
Lewis et al, 2016 [42]	1	63	Male	TrV 1 (100)	*Enterococcus faecium* 1 (100)	NR 1 (100)	Clinical cure 0 (0)<br>Overall mortality 1 (100)<br>IE-specific mortality 1 (100)

Blackwood et al, 2017 [43]	1	4	Female	NR 1 (100)	*Staphylococcus aureus* 1 (100)	NR 1 (100)	Clinical cure 1 (100)<br>Overall mortality 0 (0)
Alsobayeg et al, 2018 [44]	1	56	Female	AoV 1 (100)<br>MV 1 (100)<br>TrV 1 (100)	*Aspergillus flavus* 1 (100)	Amphotericin B 1 (100)<br>Surgical management 1 (100)	Clinical cure 0 (0)<br>Deaths overall 1 (100)<br>IE-specific mortality 1 (100)
Kim et al, 2018 [45]	1	61	Male	Mural 1 (100)	*Aspergillus* spp. 1 (100)	Voriconazole 1 (100)	Clinical cure 0 (0)<br>Overall

							mortality 1 (100) IE-specific mortality 1 (100)
Albuquerque et al, 2019 [6]	1	62	Male	AoV 1 (100)	*Proteus mirabilis* 1 (100)	Aminopenicillin 1 (100) Antistaphylococcal penicillin 1 (100) Antipseudomonal penicillin 1 (100) Surgical management 1 (100)	Clinical cure 1 (100) Overall mortality 0 (0)

Abbo et al, 2019 [46]	1	68	Male	MV 1 (100)	*Enterococcus faecium* 1 (100)	Aminopenicillin 1 (100)<br>Daptomycin 1 (100)	Clinical cure 1 (100)<br>Overall mortality 0 (0)
Eid et al, 2020 [47]	1	65	Male	MV 1 (100)	*Staphylococcus epidermidis* 1 (100)	NR 1 (100)<br>Surgical management 1 (100)	Clinical cure 1 (100)<br>Overall mortality 0 (0)
Thavamani et al, 2020 [48]	1	18	Male	NR 1 (100)	*Klebsiella* spp. 1 (100)<br>*Escherichia coli* 1 (100)	NR 1 (100)	Clinical cure 1 (100)<br>Overall mortality 0 (0)

[a] Defined as clinical resolution of the infection as a result of treatment.

AoV: aortic valve; IE: infective endocarditis; MV: mitral valve, NR: not reported; PV: pulmonary valve; TrV: tricuspid valve
