# Supplementary material for: Endocarditis in Liver Transplant Recipients: A Systematic Review
Source: J Clin Med. 2021 Jun 16;10(12):2660. doi: 10.3390/jcm10122660 (PMC8235265; doi:10.3390/jcm10122660)
Supplement: Supplementary file 1 [file jcm-10-02660-s001.zip › jcm-1252185-supplementary/@Supplementary Table 2.pdf]

Supplementary Table 2. Microbiology of Infective Endocarditis in liver transplant recipients. Values show cases among patients with available data.

Characteristic	Value
**Gram-positives, n (%)**	**43/62 (69.4%)**
Enterococcus, n (%)	16/62 (25.8%)
Enterococcus faecalis, n (%)	9/16 (56.3%)
Enterococcus faecium, n (%)	5/16 (31.3%)
Staphylococcus aureus, n (%)	14/62 (22.6%)
Coagulase-negative *Staphylococcus*, n (%)	8/62 (12.9%)
Streptococcus spp., n (%)	2/62 (3.2%)
**Gram-negatives, n (%)**	**6/62 (9.7%)**
Klebsiella spp., n (%)*	3/62 (4.8%)
Escherichia coli, n (%)*	2/62 (3.2%)
Proteus mirabilis, n (%)	1/62 (1.6%)
Enterobacter spp., n (%)	1/62 (1.6%)
**Fungi, n (%)**	**16/62 (25.8%)**
Aspergillus spp., n (%)	13/62 (21%)
Candida albicans, n (%)	1/62 (1.6%)
Pseudallescheria boydii, n (%)	1/62 (1.6%)
Phaeoacremonium parasiticum, n (%)	1/62 (1.6%)
**Polymicrobial, n (%)**	**9/62 (14.5%)**
**Culture negative, n (%)**	**5/54 (9.3%)**

* One patient had a polymicrobial infection by *Klebsiella* spp. and *E. coli*.
